# A Rare Cause of Refractory Severe Polyhydramnios: Antenatal Bartter Syndrome

**DOI:** 10.3390/medicina57030272

**Published:** 2021-03-16

**Authors:** Gina Nam, Angela Cho, Mi-Hye Park

**Affiliations:** 1 Department of Obstetrics and Gynecology, Chung-Ang University Hospital, Chung-Ang University College of Medicine, Seoul 06973, Korea; ginanam@caumc.or.kr; 2 Asan Medical Center, Department of Obstetrics and Gynecology, University of Ulsan College of Medicine, 88, Olympic-ro 43-gil, Songpa-gu, Seoul 05505, Korea; angela012687@gmail.com; 3 Department of Obstetrics and Gynecology, Ewha Womans University School of Medicine, Seoul 07804, Korea

**Keywords:** antenatal Bartter syndrome, polyhydramnios, amniotic fluid, indomethacin

## Abstract

*Background*: Antenatal Bartter syndrome is an autosomal recessive disorder causing severe polyuria that leads to severe polyhydramnios and preterm labor. Prenatal diagnosis of antenatal Bartter syndrome is difficult because the genetic diagnosis can only be confirmed following a clinical diagnosis in infants. Reports of prenatal diagnosis and treatment of antenatal Bartter syndrome are limited. *Case Presentation:* We present the case of a 33-year-old pregnant woman with refractory polyhydramnios at 31 weeks of gestation. There were no structural anomalies or placental problems on ultrasonography; therefore, antenatal Bartter syndrome was suspected. With repeated amniocentesis and indomethacin therapy, the pregnancy continued to 36 weeks of gestation. The clinical features of the infant and subsequent genetic testing confirmed the diagnosis of antenatal Bartter syndrome. The baby was in good clinical condition at the 3-month follow-up visit. *Conclusions*: For pregnant women with early onset and refractory severe polyhydramnios without morphological anomalies, antenatal Bartter syndrome should be highly suspected.

## 1. Introduction

Bartter syndrome is a severe tubulopathy causing renal salt wasting due to alterations in ion channels located in the thick ascending limb of Henle’s loop [1]. There are two distinct presentations of Bartter syndrome: antenatal Bartter syndrome and classical Bartter syndrome [2]. Bartter syndrome can be classified into five subtypes based on the underlying mutant gene: type 1, *SLC12A1*; type II, *KCNJ1*; type III *CLCNKB*; type IV, *BSND*; and type V, *CASR* [3]. Antenatal Bartter syndrome is frequently caused by mutations in the *SLC12A1* or *KCNJ1* genes [3]. Although the classic form develops in childhood, antenatal Bartter syndrome typically presents with marked fetal polyuria that leads to polyhydramnios and premature delivery [1,4]. Affected neonates are usually born premature due to polyhydramnios. Infants exhibit postnatal polyuria, vomiting, failure to thrive, hypercalciuria, and subsequent nephrocalcinosis [2]. Prenatal diagnosis is desirable to schedule appropriate management of the mother and newborn. Although biochemical analysis of amniotic fluid would be beneficial for diagnosis, studies of amniotic fluid in Bartter syndrome have yielded conflicting results [1,4,5,6,7]. Therefore, prenatal diagnosis of Bartter syndrome is difficult.

Unexplained and refractory severe polyhydramnios is a challenge for obstetricians. Antenatal Bartter syndrome is a rare cause of polyhydramnios. We herein report a case of unexplained refractory polyhydramnios strongly suspected to be antenatal Bartter syndrome and confirmed by postnatal genetic diagnosis. 

## 2. Case

**Figure 1:**
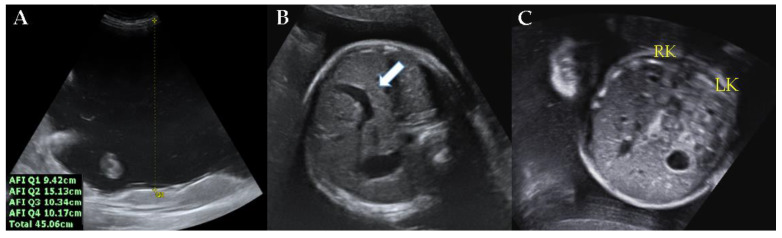
Ultrasonographic findings at 31 weeks of gestation. (**A**) Amniotic fluid index was 45.06 cm. (**B**) Persistent right umbilical vein (arrow) was observed. (**C**) There were no specific findings in either kidney of the fetus. AFI, amniotic fluid index; LK, **left** kidney; RK, **right** kidney.

A 33-year-old nulliparous woman was referred to our center at 31 weeks of gestation for severe polyhydramnios. The patient complained of dyspnea and abdominal distension. The patient had previously undergone amnioreduction twice at another center at 27 and 30 weeks of gestation. One liter of amniotic fluid was removed at each amnioreduction. There was no notable personal and family history. Physical examination revealed no tenderness in the whole abdomen, and the cervix was closed. There was no vaginal bleeding or watery discharge. The maternal oral glucose tolerance test was normal. At the time of the visit to our center, the amniotic fluid index was found to be 45.06 cm via ultrasonography (Figure 1A). The estimated fetal weight was equivalent to 31 weeks of gestation. A persistent right umbilical vein was observed (Figure 1B). Neither kidney exhibited abnormal findings (Figure 1C). There were no placental abnormalities.

At our center, amnioreduction was performed three times, with a total drainage volume of 8.4 L (2.5 L, 3 L, and 2.9 L at 31, 33, and 35 weeks of gestation, respectively). Karyotyping, performed during the first drainage of the amniotic fluid, revealed no specific chromosomal abnormalities. Despite the repeated amnioreduction every 2 weeks and indomethacin therapy because of the presumptive diagnosis of antenatal Bartter syndrome, the patient complained of aggravating dyspnea and abdominal distension. Elective surgery was scheduled at 36+1 weeks of gestation. A 2.21 kg (approximately 25th percentile) male infant was delivered via cesarean section. The 1 and 5 min Apgar scores were 9 and 10, respectively.

**Figure 2:**
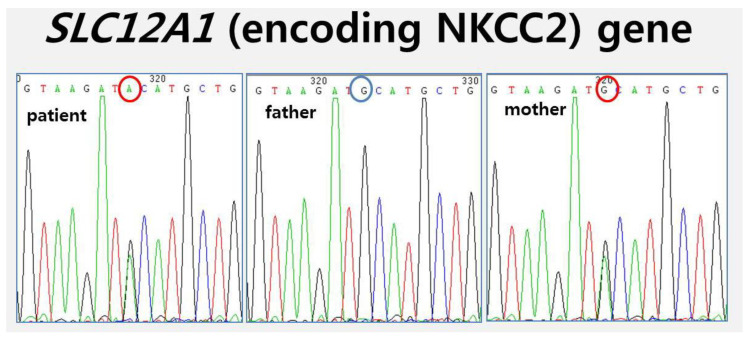
NKCC2 protein mutation sequencing revealed novel pathogenic mutations in the *SLC12A1* gene, which was implicated in antenatal type I Bartter syndrome.

We performed genetic testing of the infant; the final result was Bartter syndrome with a type I mutation in *SLC12A1* (encoding NKCC2). The infant had a heterozygous c.560G>A in exon 3 (p.Cys(TGC)187Tyr(TAC)), which was inherited from the mother on a *SLC12A1* (encoding NKCC2) gene mutation (Figure 2). Hyponatremia, hypokalemia, and elevation of plasma renin and aldosterone levels were observed in the newborn. In addition, the infant exhibited signs of polyuria. At 3-months follow-up, the baby was alive, asymptomatic, and in good clinical condition with supportive care.

## 3. Discussion

Polyhydramnios is diagnosed when the amniotic fluid index is greater than or equal to 24 or 25 cm, which is the 95th or 97.5th percentile, respectively, in normal singleton pregnancies [8]. It is caused by congenital anomalies and genetic disorders of the fetus, multiple gestations, maternal diabetes, placental anomalies, and fetal anemia [9]. The fetus and placenta should be examined with detailed ultrasonography scanning to differentiate from anomalies, such as anencephaly, cleft palate, tracheoesophageal fistula, diaphragmatic hernia, and placental chorioangioma [10]. Maternal investigation is also necessary for the diagnosis of potential maternal diabetes [11]. 

Antenatal Bartter syndrome is a rare cause of early onset and refractory severe polyhydramnios during the second trimester, without the presence of associated congenital anomalies [1]. Bartter syndrome is an autosomal recessive inherited rare disorder [12], characterized by hypokalemia, metabolic alkalosis, and secondary hyperaldosteronism [2]. It is caused by mutations in the genes involved in sodium chloride transport in the thick ascending limb of Henle’s loop and in the distal convoluted tubule [5]. Antenatal Bartter syndrome is classified into type I and type II based on the underlying mutant *SLC12A1* and *KCNJ1* genes [3]. Our patient had an *SLC12A1* gene mutation and was diagnosed with antenatal Bartter syndrome. The *SLC12A1* gene encodes the sodium–potassium–chloride co-transporter via the transporter named NKCC2 at the apical membrane of the thick ascending loop of Henle [13]. *SLC12A1* disruption results in hypercalciuria and severe volume depletion with early presentation [14]. Therefore, antenatal Bartter syndrome typically presents with fetal polyuria, which leads to polyhydramnios and preterm labor. 

The final diagnosis can only be made postnatally because a method of genetic diagnosis of a fetus with Bartter syndrome during pregnancy has yet to be established [1,6]. There have been several attempts to predict the disease prenatally by analyzing amniotic fluid, maternal serum, and urine. Elevated chloride levels in amniotic fluid have previously been reported for Bartter syndrome cases [1]. Matsushita et al. reported a case of Bartter syndrome with extremely decreased electrolyte levels in the maternal urine accompanied by elevated levels in the amniotic fluid [6]. However, Garnier et al. reported discordant results by analyzing the value of amniotic fluid biochemistry for the prediction of Bartter syndrome based on a series of 16 cases [5]. This series did not show any significant difference in electrolytes, such as sodium, chloride, potassium, calcium, and phosphorus, between Bartter groups and control groups [5]. Shalev et al. suggested that amniotic fluid aldosterone is a reliable marker for the prenatal diagnosis of Bartter syndrome [15]. In addition, Nakanishi et al. reported an elevated aldosterone level in the maternal blood and amniotic fluid in Bartter syndrome [7]. However, a retrospective case–control study, based on 36 cases of postnatally confirmed Bartter syndrome, showed conflicting results. There was no difference in amniotic fluid aldosterone concentrations from fetuses with Bartter syndrome and controls with or without polyhydramnios [4]. When the amniotic fluid Bartter index (AF-BI), defined as total protein (expressed in g/L) multiplied by AFP (expressed in MoM) and considered abnormal when ≤1.2, was used, three of four patients were incorrectly diagnosed with Bartter syndrome [16,17]. 

Although prenatal diagnosis of Bartter syndrome is difficult, early recognition of the disease enables maternal treatment with amnioreduction and indomethacin. Indomethacin treatment is effective in preventing polyhydramnios in Bartter syndrome cases [6]. In addition, indomethacin is known to protect against severe electrolyte imbalance and fluid loss after birth [10]. However, indomethacin may lead to constriction of the ductus arteriosus of the fetus [18]. Hence, the fetus must be closely monitored to avoid the potential harmful effects of indomethacin. 

Prenatal diagnosis of Bartter syndrome is still challenging because of the inconsistency of published data. However, prenatal suspicion of Bartter syndrome is important for several reasons. The first reason is the need to treat the early onset of polyhydramnios and take precautions regarding premature delivery. Second, prenatal suspicion of Bartter syndrome enables the preparation of appropriate neonatal support by immediate fluid and electrolyte management. Third, it is important for parental counseling regarding overall patient prognosis. We precisely suspected a diagnosis of antenatal Bartter syndrome, and the prenatal treatment was sufficient to prevent perinatal complications.

## 4. Conclusions

It is necessary to consider the possibility of antenatal Bartter syndrome when we encounter early onset and refractory polyhydramnios without evidence of congenital anomalies. The importance of appropriate treatment and postnatal genetic confirmation in antenatal Bartter syndrome cases should be emphasized. Future studies are needed to develop the biomarker for prenatal diagnosis of disease.

## Data Availability

The data presented in this study are available on request from the corresponding author. The data are not publicly available due to privacy and ethical concerns.

## References

[1] Dane B., Yayla M., Dane C., Cetin A. (2007). Prenatal diagnosis of Bartter syndrome with biochemical examination of amniotic fluid: Case report. Fetal Diagn. Ther..

[2] Bhat Y.R., Vinayaka G., Sreelakshmi K. (2012). Antenatal Bartter syndrome: A review. Int. J. Pediatr..

[3] Lee B.H., Cho H.Y., Lee H.K., Han K.H., Kang H.G., Ha I.S. (2012). Genetic basis of Bartter syndrome in Korea. Nephrol. Dial. Trans..

[4] Rachid M.L., Dreux S., Pean de Ponfilly G., Vargas-Poussou R., Czerkiewicz I., Chevenne D. (2016). Prenatal diagnosis of Bartter syndrome: Amniotic fluid aldosterone. Prenat. Diagn..

[5] Garnier A., Dreux S., Vargas-Poussou R., Oury J.F., Benachi A., Deschênes G. (2010). Bartter syndrome prenatal diagnosis based on amniotic fluid biochemical analysis. Pediatr. Res..

[6] Matsushita Y., Suzuki Y., Oya N., Kajiura S., Okajima K., Uemura O. (1999). Biochemical examination of mother’s urine is useful for prenatal diagnosis of Bartter syndrome. Prenat. Diagn..

[7] Nakanishi T., Suzumori N., Mizuno H., Suzuki K., Sato T., Tanemura M. (2005). Elevated aldosterone in amniotic fluid and maternal blood has diagnostic potential in pregnancies complicated with a fetus of Bartter syndrome. Fetal Diagn. Ther..

[8] Magann E., Sandlin A.T., Norton M. (2016). Amniotic fluid volume in fetal health and disease. Callen’s Ultrasonography in Obstetrics and Gynecology.

[9] Moore T.R., Cayle J.E. (1990). The amniotic fluid index in normal human pregnancy. Am. J. Obstet. Gynecol..

[10] Hamza A., Herr D., Solomayer E.F., Meyberg-Solomayer G. (2013). Polyhydramnios: Causes, diagnosis and therapy. Geburtshilfe Frauenheilkd.

[11] Magann E.F., Chauhan S.P., Doherty D.A., Lutgendorf M.A., Magann M.I., Morrison J.C. (2007). A review of idiopathic hydramnios and pregnancy outcomes. Obstet. Gynecol. Surv..

[12] Kurtz I., Cohen J.J., Harrington J.T., Madias N.E., Zusman C.J. (1998). Molecular pathogenesis of Bartter’s and Gitelman’s syndromes. Kidney Int..

[13] Wongsaengsak S., Vidmar A.P., Addala A., Kamil E.S., Sequeira P., Fass B., Pitukcheewanont P. (2017). A novel SLC12A1 gene mutation associated with hyperparathyroidism, hypercalcemia, nephrogenic diabetes insipidus, and nephrocalcinosis in four patients. Bone.

[14] Mrad F.C.C., Soares S.B.M., de Menezes Silva L.A.W., dos Anjos Menezes P.V., Simões-e-Silva A.C. (2020). Bartter’s syndrome: Clinical findings, genetic causes and therapeutic approach. World J. Pediatr..

[15] Shalev H., Ohaly M., Meizner I., Carmi R. (1994). Prenatal diagnosis of Bartter syndrome. Prenat. Diagn..

[16] Allaf B., Dreux S., Schmitz T., Czerkiewicz I., Le Vaillant C., Benachi A., Houfflin-Debarge V., Maréchaud M., Oury J.F., Muller F. (2015). Amniotic fluid biochemistry in isolated polyhydramnios: A series of 464 cases. Prenat. Diagn..

[17] Konrad M., Nijenhuis T., Ariceta G., Bertholet-Thomas A., Calo L.A., Capasso G., Emma F., Schlingmann K.P., Singh M., Trepiccione F. (2021). Diagnosis and management of Bartter syndrome: Executive summary of the consensus and recommendations from the European Rare Kidney Disease Reference Network Working Group for Tubular Disorders. Kidney Int..

[18] Van Overmeire B., Van de Broek H., Van Laer P., Weyler J., Vanhaesebrouck P. (2001). Early versus late indomethacin treatment for patent ductus arteriosus in premature infants with respiratory distress syndrome. J. Pediatr..

